# Supplementary material for: Content-rich biological network constructed by mining PubMed abstracts
Source: BMC Bioinformatics. 2004 Oct 8;5:147. doi: 10.1186/1471-2105-5-147 (PMC528731; doi:10.1186/1471-2105-5-147)
Supplement: Additional File 5 — The original Chilibot query results of the term "long-term potentiation (LTP)" and 22 other terms, limiting the latest references analyzed to the years 1990, 1995, 2000, and 2004. [file 1471-2105-5-147-S5.bz2 › chilibotAdditionalFile5/ltp2004/html/ZIF268_CAMKIV.html]

 


 **ZIF268** and **CAMKIV** 
  
Found 1 abstracts in PubMed,  **1 abstracts were retrieved and analyzed**.  


---

 Search Google  |
 PDF files only 
|  EDU domain only 

---

- J Neurochem, 2002   To investigate regulation of D2 receptor gene expression by protein kinases, we evaluated effects of constitutively active MAPK kinase kinase , Ca2ion calmodulin dependent protein kinase II, **CaMKIV** and cyclic AMP dependent protein kinase on D2R promoter activity using luciferase reporter gene assays.
  A 1.5 kbp fragment containing the rat D2R promoter was cloned upstream of the reporter and transfected into D2R expressing NB2A cells or nonexpressing NG108 15 and C6 glioma cells.
  MEKK and CaMKII, but not **CaMKIV** and PKA, increased promoter activity 4.5 and 1.5 fold, respectively, in NB2A cells.
  Inhibitory effects of a MEK inhibitor and lack of effect by dominant negative JNK1 or DN p38MAPK revealed that ERK but not JNK and p38MAPK is involved in MEKK induced promoter activation.
  Deletion and mutation of the promoter revealed that the MEKK responsive region was Sp1 site B between nucleotides 56 and 47.
  Overexpression of Sp1 suppressed promoter activity without affecting MEKK induced activation.
  Interestingly, overexpression of **Zif268** increased promoter activity through the region between nucleotides 56 and 36.
  Increased activity by **Zif268** was additive with CaMKII induced activation but not with activation induced by MEKK.
  Co transfection with CaMKII stimulated nuclear translocation of **Zif268**.
  These results suggest that ERK and CaMKII positively regulate the D2R promoter.
  **Zif268** is a potential transcription factor for the CaMKII dependent pathway.
